# Vertical Equity in Healthcare Financing: A Progressivity Analysis for the Italian Regions

**DOI:** 10.3390/healthcare10030449

**Published:** 2022-02-26

**Authors:** Guido Citoni, Domenico De Matteis, Margherita Giannoni

**Affiliations:** 1 Department of Molecular Medicine, Sapienza University of Rome, 00161 Rome, Italy; guido.citoni@uniroma1.it; 2 Istituto Nazionale della Previdenza Sociale (INPS), 35131 Padua, Italy; dematteisdomenico@hotmail.com; 3 Department of Economics, University of Perugia, 06123 Perugia, Italy; 4 Institute of Management, Scuola Superiore S. Anna Pisa, 56127 Pisa, Italy

**Keywords:** equity in healthcare financing, progressivity, vertical equity

## Abstract

Background: The aim of this paper is to measure for the first time in Italy the progressivity of healthcare financing systems at the regional level by using the Kakwani index (KI), the most widely used summary measure of progressivity in the healthcare financing literature. Methods: KIs were reported by region and by health financing sources for the year 2015. Results: There were significant vertical inequities in healthcare financing at both national and regional level. OOP (out-of-pocket) payments and value added tax were slightly regressive; income taxation on firms and households was progressive. Conclusions: After the introduction of fiscal federalism during the 90s, the healthcare financing system became regressive. A regional divide emerged: Overall regressivity is higher in the south and lower in the north, partly compensated by the interregional equalization mechanism, based on the redistribution of VAT from northern to southern regions. In times of policy interventions aiming at recovering the economy during the COVID-19 pandemic, it is important to monitor equity in healthcare financing.

## 1. Introduction

Progressivity is the usual way of measuring vertical equity in healthcare financing [1]. Previous comparative analyses of European Countries estimated vertical equity in healthcare financing as a weighted average of the Kakwani indices [2] of financing sources, where the weights are equal to the proportion of total payments accounted for by each source [1,3]. Previous evidence for Italy obtained by using cross-sectional data from 1987–1992 showed that it was a progressive system, and that it was the European country with the lowest degree of progressivity [1,3]. Another comparative study by Wagstaff et al. [4] on some European countries including Italy was based on cross-sectional data for the period 1987–1993. Direct taxes and social security contributions were the most progressive sources of financing in Italy; regressive sources were direct payments and indirect taxes. Italy showed levels of vertical equity in public financing close to other European countries with a similar health care financing system, such as the UK [4]. Compared to previous analyses, the general healthcare financing system revealed a higher degree of progressivity for Italy [4]. With the introduction of fiscal federalism in healthcare financing in the late nineties [5,6,7] the system became heavily decentralized at the regional level, with varying financing rules across regions, particularly in terms of co-payments for public healthcare services, such as drugs, specialists, and diagnostic care [8,9]. This has affected the vertical equity of the system. Rafaniello and Spandonaro showed that in Italy during the nineties, OOP (out-of-pocket) spending was significantly regressive; among the sources of public health financing, VAT (value added tax) was highly regressive, whereas both direct taxation and social security contributions appeared progressive [10]. The latter were replaced in 1996 by regional corporate tax (IRAP—*Imposta Regionale sulle Attività produttive*), a tax on the value of net production deriving from the usual exercise of activities aimed at the production or exchange of goods and services, roughly corresponding to the sum of wages and profits), which was shown to be progressive as well. Differently from previous analyses, Italy showed a slightly regressive healthcare financing system [10]. At the beginning of this millennium, Italy was ranked by the WHO as the second most equitable system in the world after France [11]. Moreover, after the introduction of fiscal federalism, there was mounting evidence that regional decentralization in healthcare delivery and financing was followed by an increase in horizontal income-related inequity in health and healthcare utilization at the regional level [12,13,14]. In Italy, there is a deep socio-economic north–south divide, with the northern regions showing the highest levels of per-capita GDP (online resource 1: Figure S1) [6]. However, there is no evidence on progressivity at the regional level for Italy, as previous analyses were focused only on the national level. So far, regional differences in financing and in progressivity have been addressed in various studies, mainly for health systems where geographical dimensions of regions were important, such as in China [15] or for other countries—but not for Italy—where there was a deep decentralization in the structure and organization of regional systems [16,17,18].

The aim of this paper was to measure the progressivity of the Italian healthcare financing system at the regional level. This work fills the gap with the first comprehensive analysis of progressivity in Italian regions. We measured progressivity as a departure from proportionality in the relationship between payments toward the provision of healthcare and ATP (ability to pay) [19,20] by using the Kakwani index methodology for the year 2015 [2]. We used households’ gross income as a proxy for ATP. We believe that income gross of taxes is a good benchmark for measuring the impact of health financing on the distribution of income: Any inference about the distributional impact of health financing should measure ATP gross of all health care, tax, and employees’ social insurance payments [19]. Our research questions are the following: To what extent are payments for healthcare related to ability to pay in (a) Italy and (b) its main areas and regions? Is the relationship at the overall system level still progressive or has it changed over time? Are there variations between regions within Italy? Is the public interregional redistribution of revenues (equalization) dampening or accentuating such variations?

The paper is structured as follows. Section 2 describes the main sources of healthcare financing in Italian regions. The data and methods used are discussed in Section 3. Results at both the national and regional levels are reported and discussed with limitations in Section 4. Section 5 highlights the implications and Section 6 offers conclusions.

## 2. Sources of Healthcare Financing in Italy

There are four main sources of healthcare financing in Italy to be considered: regional direct taxes, indirect taxes set at the national level (VAT), private insurance, and OOP (out-of-pocket) payments [6]. Regional direct taxes are the following: 1—corporate tax (IRAP); 2—personal income tax (a surcharge on income tax, called *Addizionale IRPEF—Imposta sui Redditi delle Persone Fisiche*). In 2015, public sources represented 73.4% of total financing (IRAP 14.8%, IRPEF 6.3%, VAT 52.3%), while the private share was 26.6 (24% OOP and 2.6% private insurance) (online resource 1: Figure S2). Excise duties on petrol were present in the original approach of federalism (see Legislative Decree 56/00); subsequently, in 2013, they were canceled with the new fiscal federalism [6]. The health financing structure was accompanied by an interregional tax equalization mechanism, based on a redistribution of VAT revenues among regions. We checked if this process has affected the overall progressivity/regressivity level of the health financing system. The annual quota of resources that belongs to each region to provide essential levels of care (LEA) is determined with a mixed system, partly based on a capitation formula adjusted for population needs [20]. The actual mechanism of resource allocation is mainly the result of contractual agreements among regions. This is quite different from that envisaged at the beginning of the regionalization process after 1996, which instead was aimed at fully compensating regional differences in population needs by taking into account regional variations in fiscal capacity—systematically higher in northern regions that are richer than the others—and in costs of policymaking that particularly smaller regions face [21,22,23]. 

Regional shares of health financing sources are variable among regions. Details on the shares of the main sources of healthcare expenditure financing in 2015 derived from official health accounts estimates are reported in supplementary resources (Online resource 1: Table S1). At the national level, over 50% of financing comes from indirect taxes. There are wide differences between regions. In most southern regions, the share of indirect taxes is more than 70%, while the share of direct taxation is higher among the richest regions in the North (online resource 1: Table S1). Importantly, southern regions’ financing is highly based on the redistribution of public funds (VAT revenues) from the northern regions. We discuss the direction of such redistribution, and check if it acts the right way by reducing the overall level of regressivity of financing. Another distinguishing feature of the health financing systems is the balance between public pre-payments and OOP payments in a context of high regional variations. For example, the Aosta Valley Region obtains about one-third of its funding for healthcare from OOP payments, while Campania obtains only 18% (online resource 1). Private insurance plays a relatively minor role in most regions, with an average share of 2.6%. Its contribution is non-negligible only in Lombardy (9.2%), Lazio (5.1%), and Veneto (3.6%) (online resource 1: Table S1). 

## 3. Materials and Methods

Two types of data are required for the progressivity analysis: survey data, in order to establish the distribution of payments across households, and aggregate data, in order to determine the macro-weights to be assigned to each financing source. The most suitable source of survey data is a household income and expenditure survey, which should contain good data on the two central variables: payments toward healthcare and the ability to pay [24]. Unfortunately, no such comprehensive survey is available for Italy, so we performed a statistical matching between two datasets (details and main statistics are provided in online resource 2). First, the survey data to establish the distribution of private expenditures for healthcare and other goods across households by using cross-sectional data from the Italian National Institute of Statistics (ISTAT) Household Budget Survey (HBS *n* = 15,013 households) for 2015 [25] and second, households’ income at the micro level, by using pre- and post-tax income Eurostat EU-SILC microdata (*n* = 17,985 households) [26]. Private insurance (PI) includes individual private health insurance (data on supplementary group insurances were not available); OOP expenditures were directly calculated from the 2015 HBS. A macroeconomic coherence test was also performed (online resource 2 reports on the above procedures). There are three distinct stages for an analysis of progressivity [2,24]: first, to establish the progressivity of each source of finance: direct taxes, indirect taxes, out-of-pocket, and private health insurance; second, to define, for each region the weight of each source of financing, in order to define the financing mix; and third, to establish overall progressivity for each region using the financing mix. In this way, we could compare the indices for all regions [2,24].

We used the Kakwani index (KI) [2] methodology that is the most widely used summary measure of progressivity in both the tax and the healthcare financing literature; this index considers that a tax system can deviate from proportionality: Being an aggregate index, it gives a good summary view of progressivity but it conceals specific peculiar effects and distributive patterns [1,4,24]. The index was measured as twice the area between a payment concentration curve and the Lorenz curve for income, and calculated as:

KI = C − G(1)

where C is the concentration index for health payments, and G is the Gini coefficient of the income variable. The G is always positive by construction and varies between 0 and 1. The concentration index C varies between −1 and +1, depending on whether the tax seriously affects the taxpayers who are poorer or those who are richer, and the value of KI varies from −2 to 1. A negative value indicates regressivity, while a positive value indicates progressivity and 0 proportionality [2].

## 4. Results

Firstly, we estimated the concentration index (C) and the Gini coefficient (G) and the Kakwani index (KI) for each source of financing in Italy (Table 1).

All estimated indexes were significantly different from zero for all sources of financing, with the only exception of KI estimates for private insurance (Table 1). The latter could be due to a low proportion of individually insured individuals in the sample and to the fact that those covered by supplementary group insurance were not included in the data. We also estimated the concentration curves for each source of financing in Italy as well as the Lorenz curve for incomes (not reported here and available upon request from the authors). The concentration curves for IRAP and IRPEF surcharge lie outside the Lorenz curve, suggesting that these are progressive sources of financing. VAT and OOP appear to lie inside the Lorenz curve; therefore, they are regressive sources of finance. The curve for private insurance appears to lie outside the Lorenz curve at lower ATP but inside it at very high ATP: such a crossing between the concentration curve and Lorenz curve can be due to different levels of risk aversion and propensity to buy private health insurance between the richest and the poorest in society. We decided, though, to use their values in the calculation of the aggregate KI, as the choice leaves the index substantially unaffected given the negligible share of insurance payments in the financing mix.

**Figure 1:**
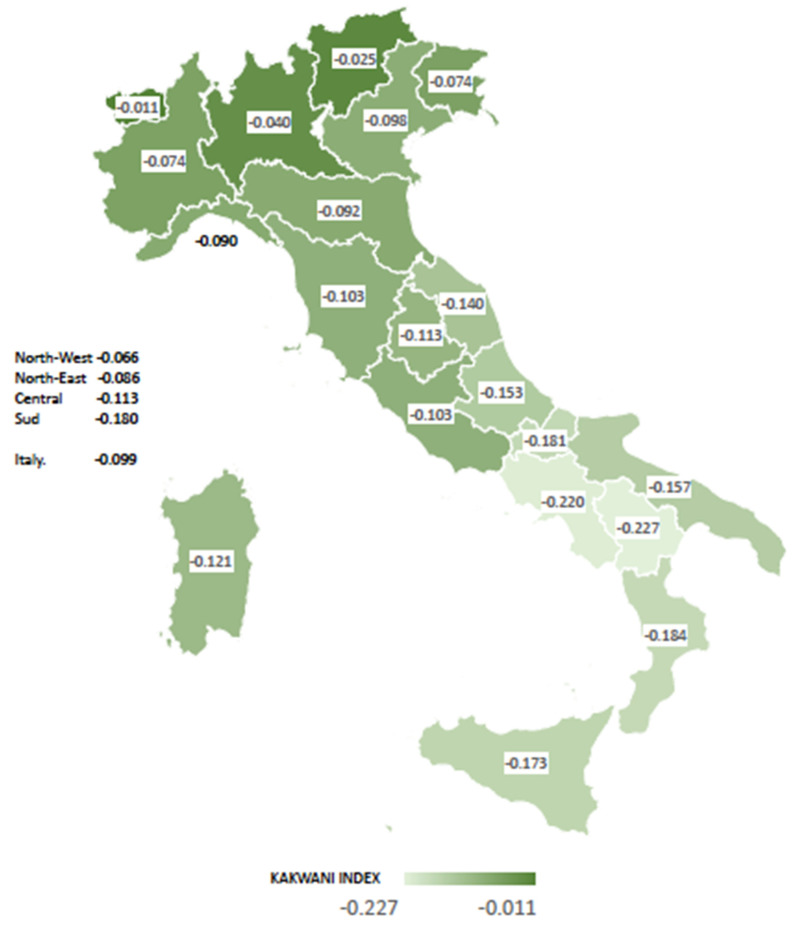
Overall progressivity at national and regional levels—Italy, 2015 (regional weighted Kakwani Index). Source: Authors’ estimates based on Italian National Institute of Statistics household budget survey [25] and IT-SILC survey data [26]. Weighted Kakwani indices. All results are statistically different from zero at the 5% level of significance.

We then estimated progressivity at the regional level by using the KI approach. Figure 1 shows the overall progressivity levels estimated at the National and the Regional level. Table A1 shows the detailed regional estimates of KIs for the main sources of financing, together with their standard errors, *p*-values, and confidence intervals. 

There is regressivity at the national level (KI = −0.099) as well as in all regions (Figure 1, Table A1). Two Southern Italian regions (Campania and Basilicata) showed the highest regressivity (KI = −0.22), while the lowest KIs were found in the northern area in Aosta Valley (KI = −0.011) and Trentino Alto Adige (KI = −0.025), very close to a proportional index. In the southern area, health financing was more regressive (KI = −0.18) than in the northern area, particularly in the northwest (KI = −0.053) (Figure 1).

VAT (Table A1) and OOP (including co-payments) (Table A2) KIs were negative in all regions, but they were more regressive in the regions of South Italy. This geographical gradient can be explained by the higher consumption to income ratio in regions of the south. Direct taxes (IRAP and IRPEF surcharges) are always progressive (Table A1). Private insurance estimates are mostly close to zero, although estimates are for almost all regions not significantly different from zero (Table A2).

Looking at the estimated KIs for the four main geographic areas of Italy, the direct taxes (IRAP and IRPEF surcharge) are everywhere progressive, while the indirect taxation (VAT) is always regressive (Table A1). OOP payments are everywhere regressive, but in South Italy they appear to be much more regressive than in the north; PI is mostly not significantly different from zero (Table A2).

We then re-estimated the progressivity of three public financing sources after their redistribution in order to understand if the interregional tax equalization mechanism—based on a redistribution of VAT revenues among regions—might have affected the global regressivity of public financing (Table A3). As mentioned above, an interregional solidarity mechanism allows regions with needs higher than their own fiscal revenues, to draw the difference from a special equalization fund, which is fed by the northern richer regions with VAT surplus. Aggregate KI of public sources went from −0.09 before equalization (ex *ante*) to a close to proportionality −0.024 after equalization (*ex post*), thus making the Italian system less regressive (Table A3). This result is due to the redistribution from the northern region’s VAT (less regressive) revenues to the southern regions (more regressive). The redistribution of VAT reduces regressivity but does not cancel it.

A full analysis of the causes of differences in financing is beyond the scope of our work. Among the social, cultural, organizational, and economic factors leading to inequity at least three require a tentative screening. Is there a bias in the progressivity of healthcare financing in southern regions, confirming the existence of the north–south divide, called “*Questione meridionale*”? Alternatively, could the differences between regions be associated with differences in the regional population size? Could these differences depend on other features such as the special autonomy status of some regions?

In our study, we had a very regressive KI, particularly for the southern regions. This result was mainly driven by their specific financing mix which is heavily based on the most regressive sources (VAT). It should also be noted that in Italy redistribution of resources from the richest regions of the north to the poorest regions of the south is also done through VAT. The specific regressivity of the source is then mitigated by the fact that VAT regressivity has a north–south gradient.

Results showed that regressivity levels were not associated with population size. Regions with fewer inhabitants can be similar to highly populated regions in the overall degree of progressivity. For example, in the north, Piedmont, and Friuli Venezia Giulia (FVG) show similar values of the KI (Figure 1), but the total population is much higher in Piedmont than in FVG (see also online resource 1: Figure S1). We noticed that most regions that were granted a “special autonomy status” showed lower levels of regressivity. These are Aosta Valley and Trentino Alto-Adige in the north and the two islands (Sicily and Sardinia) in the south. We cannot ascertain if higher autonomy could be linked to lower regressivity because these regions receive more public funding than the others. This issue, though, deserves more careful scrutiny. Some other limitations remain. First, in our study we merged two datasets. However, after the usual checks for goodness of merging procedure were applied, the bias for merged cases was significantly reduced. As discussed, KI estimates for private insurance were mostly not significant at the regional level. This could reflect the fact that there was a low proportion of individually insured individuals in the sample and those covered by supplementary group insurance were not included in the data. We decided, though, to use their values in the calculation of the aggregate KI, as the choice leaves the index substantially unaffected given the overall PI negligible share of insurance payments in the financing mix.

## 5. Discussion

The Italian system shows a high interregional variability in health financing. This heterogeneity derives from differences in tax rates, in the contributory capacity of citizens, in their share of OOP expenditure—both for private care and for public healthcare services (copayments for drugs, specialists, and diagnostic treatments)—and in the use of private insurance.

The health financing system, originally progressive, has become regressive. This result was expected, in line with previous Italian research [10], and because of the shift, over time, from direct to indirect taxation as the main source of public financing. The public revenues’ interregional equalization process from the richer northern regions to the poorer southern regions partly reduces the regressivity of the system. Aggregate KI of public sources went from −0.09 before equalization (*ex ante*) to a close to proportionality −0.024 after equalization (*ex post*).

Moreover, the study shows that aggregate results conceal different results at the disaggregated level: Regressivity was much higher in the southern than in the northern regions.

There is the need for careful monitoring of the public/private financing mix as well as of the regressivity of each public source of financing. After the COVID-19 pandemic outbreak, the focus is on measures supporting firms and consumers that could either negatively (IRAP abolishment) or positively (VAT rate reduction) impact on equity. With the post-COVID-19 pandemic outbreak economic crisis and with a geographical distribution of privately insured people concentrated in the richer northern areas, the relative disadvantage of the southern regions could increase. Moreover, as incomes and GDP fall, the relative share in financing of the most regressive source (VAT) may increase.

Some other aspects of financing need to be investigated in the future, such as catastrophic health expenditure and the redistributive effects of financing, where geographical gradients are expected as well.

## 6. Conclusions

Investing more in public healthcare with a higher weight given to the progressive public sources of financing could avoid a further reduction in vertical equity. It is important to monitor vertical equity at both national and regional levels.

## Figures and Tables

**Table 1:** Income concentration, Gini coefficient, and Kakwani index by sources of financing in Italy (2015).

(G) Gini Index by Source of Financing	Coeff.	Std.Err.	*p* > |z|	[95% Conf. Interval]	
Personal income tax (IRPEF) regional surcharge	0.382	0.004	0.000	0.375	0.390
Corporate regional tax (IRAP)	0.425	0.006	0.000	0.413	0.437
Value added tax (VAT)	0.195	0.005	0.000	0.186	0.204
Private insurance	0.365	0.053	0.000	0.261	0.469
Out of pocket (OOP)	0.214	0.009	0.000	0.196	0.232
(C) Income concentration	0.348	0.003	0.000	0.343	0.353
(K = G − C) Kakwani index by source of financing:					
Income tax (IRPEF) regional surcharge	0.035	0.004	0.000	0.026	0.043
Corporate regional tax (IRAP)	0.077	0.010	0.000	0.057	0.097
Value added tax (VAT)	−0.153	0.007	0.000	−0.166	−0.140
Private insurance	0.017	0.064	0.788	−0.109	0.144
Out of pocket (OOP)	−0.137	0.012	0.000	−0.161	−0.114

Source: Authors estimates based on Italian National Institute of Statistics household budget survey and IT-SILC survey data from Eurostat [25,26].

## Data Availability

Not applicable.

## Supplementary Materials

The following supporting information can be downloaded at: https://www.mdpi.com/article/10.3390/healthcare10030449/s1, Online Resource 1: Figure S1: Italian areas, regions and regional population density in 2015; Figure S2: Per capita GDP in Italian regions in 2015; Table S1: The Health Financing mix in Italian regions in 2015; Online Resource 2: Sources of health financing data, macroeconomic coherence check and results from the matching procedure [21,22,23,25,26,27,28,29,30,31,32].

## Appendix A

**Table A1:** Regional and national progressivity indices: Kakwani index (KI) by main public sources of health financing.

Area/Region	IRAP					IRPEF Surcharge					VAT				
	Kakwani	Std.Err	*p*	[95% Conf. Interval]		Kakwani	Std.Err	*p*	[95% Conf. Interval]		Kakwani	Std.Err	*p*	[95% Conf. Interval]	
*North West*	0.096	0.018	0.00	0.061	0.131	0.029	0.008	0.00	0.013	0.045	−0.107	0.014	0.00	−0.135	−0.079
Piedmont	0.089	0.024	0.00	0.042	0.136	0.041	0.012	0.00	0.018	0.064	−0.135	0.022	0.00	−0.178	−0.092
Aosta valley	0.117	0.037	0.00	0.044	0.191	0.054	0.016	0.00	0.024	0.085	−0.067	0.030	0.03	−0.126	−0.008
Lombardy	0.093	0.025	0.00	0.044	0.142	0.016	0.011	0.15	−0.006	0.037	−0.098	0.020	0.00	−0.137	−0.059
Liguria	0.141	0.042	0.00	0.059	0.223	0.064	0.023	0.01	0.018	0.110	−0.138	0.027	0.00	−0.191	−0.084
*North East Area:*	0.064	0.015	0.00	0.034	0.094	0.032	0.007	0.00	0.019	0.045	−0.146	0.011	0.00	−0.168	−0.125
Trentino Alto Adige	0.079	0.044	0.07	−0.006	0.165	0.028	0.019	0.14	−0.009	0.065	−0.087	0.035	0.01	−0.155	−0.019
Veneto	0.072	0.024	0.00	0.026	0.119	0.025	0.009	0.00	0.008	0.041	−0.152	0.018	0.00	−0.187	−0.117
Friuli Venezia Giulia	0.057	0.030	0.06	−0.002	0.116	0.020	0.012	0.11	−0.004	0.044	−0.116	0.018	0.00	−0.153	−0.080
Emilia Romagna	0.053	0.026	0.04	0.002	0.104	0.044	0.013	0.00	0.019	0.070	−0.158	0.018	0.00	−0.194	−0.123
*Central Area:*	0.059	0.020	0.00	0.020	0.099	0.044	0.011	0.00	0.024	0.065	−0.172	0.016	0.00	−0.203	−0.140
Toscana	0.040	0.036	0.27	−0.031	0.110	0.040	0.016	0.01	0.010	0.071	−0.162	0.021	0.00	−0.203	−0.122
Umbria	0.091	0.059	0.12	−0.025	0.207	0.047	0.028	0.09	−0.007	0.102	−0.163	0.032	0.00	−0.226	−0.100
Marche	0.070	0.031	0.02	0.009	0.131	0.052	0.017	0.00	0.018	0.086	−0.193	0.023	0.00	−0.239	−0.147
Lazio	0.065	0.031	0.03	0.005	0.125	0.044	0.017	0.01	0.010	0.078	−0.171	0.027	0.00	−0.225	−0.117
Abruzzo	0.140	0.054	0.01	0.033	0.247	0.046	0.018	0.01	0.010	0.083	−0.213	0.025	0.00	−0.263	−0.163
*South Area:*	0.098	0.024	0.00	0.051	0.144	0.052	0.007	0.00	0.039	0.066	−0.218	0.010	0.00	−0.237	−0.198
Molise	0.022	0.050	0.65	−0.075	0.120	0.027	0.023	0.24	−0.019	0.073	−0.208	0.037	0.00	−0.281	−0.135
Campania	0.107	0.042	0.01	0.025	0.189	0.037	0.014	0.01	0.009	0.066	−0.251	0.019	0.00	−0.287	−0.214
Apulia	0.073	0.056	0.19	−0.036	0.183	0.049	0.013	0.00	0.024	0.073	−0.192	0.021	0.00	−0.233	−0.152
Basilicata	0.100	0.055	0.07	−0.007	0.207	0.076	0.020	0.00	0.037	0.115	−0.243	0.027	0.00	−0.296	−0.191
Calabria	0.098	0.065	0.13	−0.030	0.226	0.057	0.015	0.00	0.028	0.087	−0.211	0.024	0.00	−0.259	−0.164
Sicily	0.102	0.52	0.05	0.001	0.204	0.068	0.017	0.00	0.035	0.102	−0.225	0.025	0.00	−0.273	−0.176
Sardinia	0.103	0.116	0.38	−0.125	0.331	0.062	0.022	0.01	0.019	0.104	−0.179	0.036	0.00	−0.249	−0.109
*Italy*	0.077	0.01	0.00	0.057	0.097	0.035	0.004	0.00	0.026	0.043	−0.153	0.007	0.00	−0.166	−0.14

Source: Authors estimates based on Italian National Institute of Statistics household budget survey and IT-SILC survey data from Eurostat [25,26].

**Table A2:** Regional and national progressivity indices: Kakwani index (KI) by private sources of health financing.

Area/Region	Out of Pocket Kakwani	Std.Err	*p*	[95% Conf. Interval]		Private Insurance Kakwani	Std.Err	*p*	[95% Conf. Interval]	
*North West Area:*	−0.091	0.025	0.00	−0.140	−0.042	0.012	0.167	0.94	−0.315	0.338
Piedmont	−0.102	0.044	0.02	−0.189	−0.015	0.114	0.153	0.46	−0.187	0.414
Aosta valley	−0.039	0.074	0.60	−0.184	0.106	−0.099	0.145	0.49	−0.383	0.185
Lombardy	−0.086	0.033	0.01	−0.151	−0.022	−0.040	0.275	0.88	−0.579	0.499
Liguria	−0.161	0.045	0.00	−0.248	−0.074	0.093	0.228	0.68	−0.355	0.542
*North East Area:*	−0.134	0.020	0.00	−0.173	−0.096	−0.010	0.064	0.88	−0.136	0.116
Trentino Alto Adige	−0.039	0.072	0.59	−0.181	0.103	−0.054	0.157	0.73	−0.362	0.254
Veneto	−0.153	0.028	0.00	−0.209	−0.098	−0.032	0.117	0.78	−0.263	0.198
Friuli Venezia Giulia	−0.114	0.040	0.01	−0.194	−0.035	−0.006	0.137	0.97	−0.274	0.263
Emilia Romagna	−0.135	0.034	0.00	−0.202	−0.068	−0.019	0.095	0.84	−0.205	0.168
*Central Area:*	−0.156	0.027	0.00	−0.210	−0.102	−0.002	0.076	0.98	−0.151	0.148
Toscana	−0.132	0.044	0.00	−0.218	−0.046	0.110	0.141	0.44	−0.167	0.387
Umbria	−0.157	0.052	0.00	−0.260	−0.054	0.487	0.365	0.18	−0.231	1.205
Marche	−0.195	0.038	0.00	−0.269	−0.120	−0.243	0.120	0.04	−0.479	−0.007
Lazio	−0.162	0.044	0.00	−0.249	−0.075	−0.096	0.097	0.32	−0.287	0.094
Abruzzo	−0.185	0.047	0.00	−0.276	−0.094	0.158	0.304	0.60	−0.440	0.756
*South Area:*	−0.207	0.016	0.00	−0.238	−0.175	0.050	0.130	0.70	−0.204	0.304
Molise	−0.163	0.050	0.00	−0.260	−0.065	0.118	0.461	0.80	−0.788	1.024
Campania	−0.297	0.029	0.00	−0.354	−0.239	−0.158	0.212	0.46	−0.574	0.258
Apulia	−0.178	0.029	0.00	−0.235	−0.122	0.188	0.247	0.45	−0.297	0.672
Basilicata	−0.281	0.049	0.00	−0.377	−0.186	0.232	0.425	0.59	−0.603	1.067
Calabria	−0.142	0.057	0.01	−0.254	−0.031	0.501	0.671	0.46	−0.816	1.817
Sicily	−0.212	0.040	0.00	−0.290	−0.133	0.043	0.262	0.87	−0.471	0.557
Sardinia	−0.138	0.063	0.03	−0.262	−0.015	−0.435	0.398	0.28	−1.217	0.348
*Italy*	−0.137	0.012	0.00	−0.161	−0.114	0.017	0.064	0.79	−0.109	0.144

Source: Authors estimates based on Italian National Institute of Statistics household budget survey and IT-SILC survey data from Eurostat [25,26].

**Table A3:** Regional and national progressivity indices: Kakwani index by public source of health financing and relative shares of financing (2015).

	Public Sources of Health Financing						All Public Sources KAKWANI INDEX
	1-Regional Corporate Income Tax (IRAP)	%	2- Regional Surcharge on Income Tax % (Add. IRPEF)		3-VAT (Value- Added-Tax) %		
Area /Region							
*North West area:*	0.096	25.40%	0.029	10.20%	−0.107	64.40%	−0.042
Piedmont	0.089	21.20%	0.041	9.70%	−0.135	69.10%	−0.070
Aosta Valley	0.117	30.50%	0.054	10.40%	−0.067	59.10%	0.002
Lombardy	0.093	30.90%	0.016	10.90%	−0.098	58.20%	−0.027
Liguria	0.141	19.00%	0.064	9.80%	−0.138	71.20%	−0.065
*North East area:*	0.064	27.20%	0.032	10.00%	−0.146	62.80%	−0.071
Trentino Alto Adige	0.079	33.00%	0.028	10.50%	−0.087	56.50%	−0.020
Veneto	0.072	24.70%	0.025	9.50%	−0.152	65.80%	−0.080
Friuli Venezia Giulia	0.057	24.70%	0.020	9.90%	−0.116	65.40%	−0.060
Emilia Romagna	0.053	26.20%	0.044	10.20%	−0.158	63.60%	−0.082
*Central area:*	0.059	20.90%	0.044	8.90%	−0.172	70.20%	−0.104
Tuscany	0.040	22.30%	0.040	9.30%	−0.162	68.40%	−0.098
Umbria	0.091	15.00%	0.047	8.40%	−0.163	76.60%	−0.107
Marche	0.070	18.80%	0.052	8.50%	−0.193	72.70%	−0.123
Lazio	0.065	27.60%	0.044	9.30%	−0.171	63.10%	−0.086
*South area:*	0.098	7.80%	0.052	6.10%	−0.218	86.00%	−0.176
Abruzzi	0.140	12.80%	0.046	7.20%	−0.213	80.00%	−0.149
Molise	0.022	1.80%	0.027	6.50%	−0.208	91.70%	−0.189
Campania	0.107	8.40%	0.037	5.50%	−0.251	86.10%	−0.205
Apulia	0.073	8.60%	0.049	6.00%	−0.192	85.40%	−0.155
Basilicata	0.100	1.10%	0.076	6.10%	−0.243	92.80%	−0.220
Calabria	0.098	0.10%	0.057	5.30%	−0.211	94.60%	−0.197
Sicily	0.102	13.00%	0.068	5.50%	−0.225	81.50%	−0.166
Sardinia	0.103	17.00%	0.062	6.90%	−0.179	76.10%	−0.115
*ITALY (ex ante)*	**0.077**	**20.20%**	**0.035**	**8.50%**	**−0.153**	**71.30%**	**−0.090**
*ITALY (ex post)*	0.077	20.20%	0.035	8.50%	**−0.060**	71.30%	**−0.024**

Source: Authors estimates based on Italian National Institute of Statistics household budget survey and IT-SILC survey data from Eurostat [25,26]. *Ex ante* regional estimates and standard errors are reported in Table A1.
