# Supplementary material for: Vertical Equity in Healthcare Financing: A Progressivity Analysis for the Italian Regions
Source: Healthcare (Basel). 2022 Feb 26;10(3):449. doi: 10.3390/healthcare10030449 (PMC8953414; doi:10.3390/healthcare10030449)
Supplement: Supplementary file 1 [file healthcare-10-00449-s001.zip › healthcare-1585445-supplementary.pdf]

Online Resource 1 – Population, per capita GDP, and health financing mix in Italy

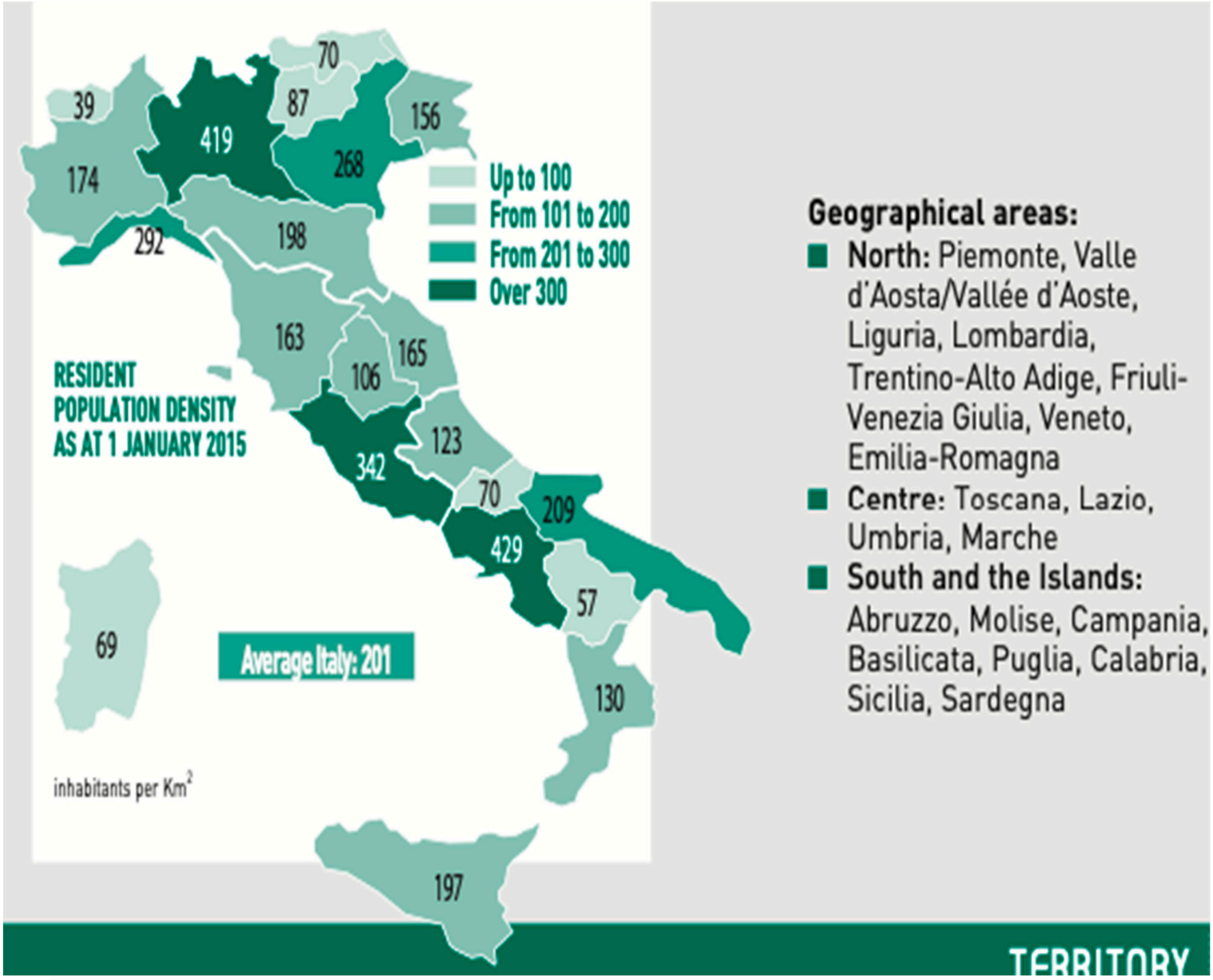

Figure S1. Italian areas, regions and regional population density in 2015. Source: Istat—Istituto Italiano di Statistica [1] p.1.

Online Resource 1 – Population, per capita GDP, and health financing mix in Italy

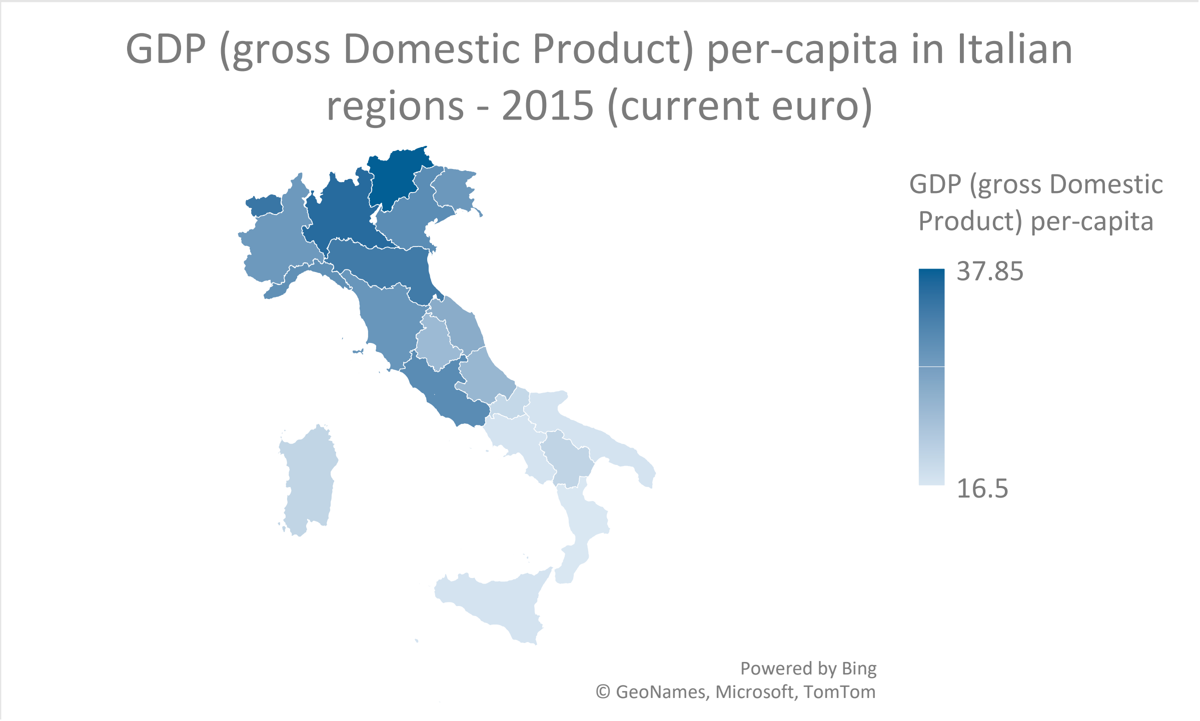

Figure S2. Per capita GDP in Italian regions in 2015. Data source: Istat—Istituto Italiano di Statistica [2]

Online Resource 1 – Population, per capita GDP, and health financing mix in Italy

Table S1. The Health Financing mix in Italian regions in 2015 (%).

Area/Region	Regional Corporate income tax (IRAP)	Regional Additional surcharge on income tax (Addizionale IRPEF)	VAT	Out-Of-Pocket	Private Insurance
North West area:	18.0%	7.2%	46.0%	26.0%	2.8%
Piedmont	15.4%	7.0%	50.0%	25.5%	2.1%
Aosta valley	20.9%	7.1%	40.6%	31.3%	0.1%
Lombardy	21.8%	7.6%	41.0%	20.4%	9.2%
Liguria	14.0%	7.2%	52.1%	26.6%	0.1%
North East area:	19.7%	7.3%	45.5%	25.5%	2.0%
Trentino Alto Adige	24.1%	7.7%	41.3%	25.9%	1.0%
Veneto	17.3%	6.6%	46.0%	26.5%	3.6%
Friuli Venezia Giulia	18.3%	7.4%	48.6%	25.6%	0.1%
Emilia Romagna	14.0%	7.2%	52.1%	26.6%	0.1%
Central area:	16.0%	6.7%	53.9%	21.3%	2.1%
Toscana	17.0%	7.0%	52.1%	22.6%	1.3%
Umbria	12.0%	6.7%	61.0%	19.7%	0.6%
Marche	14.4%	6.5%	55.8%	21.6%	1.7%
Lazio	20.3%	6.8%	46.6%	21.2%	5.1%
South area:	6.3%	4.9%	69.0%	19.0%	0.8%
Abruzzo	10.0%	5.6%	62.0%	21.4%	1.0%
Molise	1.4%	5.2%	74.2%	18.0%	1.2%
Campania	7.0%	4.5%	70.6%	17.3%	0.6%
Apulia	6.6%	4.6%	65.8%	22.2%	0.8%
Basilicata	0.9%	5.0%	76.0%	17.3%	0.8%
Calabria	0.1%	4.4%	77.0%	18.1%	0.4%
Sicily	10.4%	4.4%	65.2%	19.2%	0.8%
Sardinia	13.8%	5.6%	61.7%	18.3%	0.6%
Italy	14.8%	6.3%	52.3%	24.0%	2.6%

Data Source: Censis [3], Presidenza del Consiglio dei Ministri [4], and Corte dei Conti [5].

Online Resource 2. Sources of health financing data, macroeconomic coherence check and results from the matching procedure

A) Sources of health financing data and macroeconomic coherence check

The HBS dataset contains information on households' expenditures for goods and services according to their main social, economic, and territorial characteristics [25]. The analysis of direct and indirect taxation is based on microdata from the 2015 wave of the Italian HBS (n = 15013 households) and from EU-SILC cross-sectional wave for 2015 (n =17985 households). In order to estimate the indirect taxation component of financing, we applied a set of VAT rates to households' yearly consumption levels of goods and services subject to VAT. No attempt to correct for households' underreporting of expenditures was performed, as we relied on adjustments already performed by ISTAT. The obtained revenues were then aggregated by regions. The general taxation component was estimated as such. EU-SILC contains detailed information on households' income sources as well as on total income taxation: the last information was useless though, given that we were interested in the regional income tax surcharge (regional additional IRPEF) that is only a component of total income taxation and on IRAP, whose tax base is value-added at a firm level. Our estimation strategy started then from EU-SILC individual records: using regional tax rates, we imputed to individuals the regional income tax surcharge. No correction for income underreporting was explicitly performed, except that adopted by ISTAT. The assumption underlying IRAP imputation was that of perfect forward translation. We made the hypothesis that IRAP paid by firms would be shifted fully as increased wages to workers had the tax not been there. IRAP was then proxied by a pay-as-you-go scheme and considered as a social security contribution paid fully by wage earners and independent workers (though for them the payment is only partial because of widespread exemptions). The IRAP tax base also includes corporate profits, but our survey had profits that were not representative of the whole corporate profits, being probably underreported. This is a limitation of the data. Furthermore, even if we could have reliable estimates of profits, we would not know which IRAP rate to apply to them. Recorded profits could be obtained (and taxed) in sectors other than the main job of the income recipient (the only one we know). Therefore, we disregarded profits because they are not representative and because we do not know the sector where they are produced. The following table shows the macroeconomic coherence of our estimates obtained by assuming no fiscal evasion and by using data on revenues from the Italian Ministry of Finance; this shows an acceptable overlap.

Table S2. Macroeconomic coherence IRAP, Additional IRPEF and VAT in Italy - year 2015 (current euro).

Total Amount (Italy)	Tax base	Net tax	Estimated tax base	Estimated net tax
**IRAP**	605799341	30025499	610000000	29100000
**Additional IRPEF**	725618386	11383548	820000000	12900000
**VAT**	764156969	89641651	834000000	91400000

Data source: Italian Ministry of Finance [6].

We aggregated IRAP and Additional IRPEF by households in the EU-SILC data at household level.

B) The matching procedure

As income information, needed to measure ability to pay, was missing in HBS, this could be imputed directly from EU-SILC. The matching procedure between HBS and EU-SILC followed. We used a mixed strategy. We first used exact matching with hot ranking [7]. We used the following grouping characteristics of household head as matching variables: age group (3 classes), gender (2), educational level (5), employment condition (3), activity (2), main geographical areas (4) and whether the second member of the household earns any income (2). We ranked households, inside each cell, by total expenditure (HBS) and by disposable income (EU-SILC). We recall, though, that the above test pertains only to the last round of matching procedure (PSMATCH), because the matching procedure was done firstly in a statistical way, coupling families similar according to demographic attributes of head of family. We checked if the first stage of the procedure was good by looking at central values and variances of income for each family type in IT-SILC and in the matched file. We coupled 12647 HBS households out of 15013, with “similar” households of EU-SILC. For the unmatched 2366 households of HBS, income and tax variables by EU-SILC were imputed by using propensity score matching. This was based on the following household heads’ variables: age group (3 classes), gender (2), educational level (5), employment condition (3), activity (2), regions (20), size of the family (6) and marital status (6) [8]. The STATA Pstest command output

for testing the goodness of matching procedure showed a substantial attenuation of the distance between treated and control cases. The percentage of bias attenuation of covariates ranged from a lowest 66% to a highest 99%. PS test results are reported after the frequencies table. At the end of the two-stage procedure, all HBS households had the required variables. The final dataset contained 15013 records and all income and taxation variables needed [9]. The following table shows the frequencies of the matching variables.

Table S3. Frequencies of matching variables and output from Stata (PStest).

Survey		HBS (n=15013)		SILC (n=17985)	
**Variable**	**Description**	**Frequencies**	**%**	**Frequencies**	**%**
Age1	1= >18 and <=34 years	995	6.63	964	5.36
	2= >34 and <=64 years	8336	55.53	10099	56.15
	3= >65 years	5682	37.85	6922	38.49
Gender1	1=male	10193	67.89	11926	66.31
	2=female	4820	32.11	6059	33.69
Rip1(area)	1= North West	3284	21.87	4460	24.80
	2= North East	3382	22.53	4462	24.81
	3=Center	2791	18.59	4223	23.48
	4=South	5556	37.01	4840	26.91
Region	1=Piedmont	1112	7.41	1319	7.33
	2=Aosta Valley	480	3.20	316	1.7
	3=Lombardy	1085	7.23	1829	10.17
	4=Trentino Alto Adige	443	2.95	621	3.45
	5=Veneto	1157	7.71	1410	7.84
	6=Friuli Venezia Giulia	827	5.51	1100	6.12
	7=Liguria	607	4.04	996	5.54
	8=Emilia Romagna	955	6.36	1331	7.40
	9=Tuscany	571	3.80	1196	6.65
	10=Umbria	335	2.23	585	3.25
	11=Marche	841	5.60	1024	5.69
	12=Lazio	1044	6.95	1418	7.88
	13=Abruzzi	553	3.68	434	2.41
	14=Molise	477	3.18	260	1.45
	15=Campania	1141	7.60	1032	5.74
	16=Apulia	935	6.23	855	4.75
	17=Basilicata	555	3.70	329	1.83
	18=Calabria	724	4.82	604	3.36

Survey<br>**Variable**	**Description**	HBS (n=15013)<br>**Frequencies**	**%**	SILC (n=17985)<br>**Frequencies**	**%**
	19=Sicily	753	5.02	889	4.94
	20=Sardinia	418	2.78	437	2.43
Activity1	0=Missing			183	1.02
	1=employed	7307	48.67	8782	48.83
	2=in another condition	7706	51.33	9020	50.15
Employ1	0= Missing	1282	8.54	718	3.99
	1=Family worker and employee	10419	69.40	12801	71.18
	2=Self-employed with employees	953	6.35	1102	6.13
	3=Self-employed without employees	2359	15.71	3364	18.70
Edu1	1= Less than primary education	540	3.60	504	2.80
	2= Primary education	2902	19.33	3157	17.55
	3= Lower secondary education	4276	28.48	4680	26.02
	4= Upper secondary education	5398	35.96	6637	36.90
	5= Bachelor or equivalent	1897	12.64	3007	16.72
Staciv1	1=unmarried	2551	16.99	3055	16.99
	2=married	8252	54.97	9832	54.67
	3=separated	355	2.36	523	2.91
	4=legally separated	625	4.16	810	4.50
	5=divorced	698	4.65	798	4.44
	6=widower/ widow	2532	16.87	2967	16.50
Hsize	1=one person	4448	29.63	5244	29.16
	2=two people	4675	31.14	5390	29.97
	3=three people	2769	18.44	3552	19.75
	4=four people	2394	15.95	2919	16.23

Survey Variable	Description	HBS (n=15013) Frequencies	%	SILC (n=17985) Frequencies	%
	5=five people	546	3.64	703	3.91
	6=six or more people	181	1.21	177	0.98

Data source: Italian National Institute of Statistics household budget survey [10] and IT-SILC survey data [11].

```
pstest age_1 gender_1 activity_1 employ_1 edu_1 staciv_1 hsize regione, both graph

-----------------------------------------------------------------------------------------

                Unmatched |       Mean               %reduct |     t-test    |  V(T)/

Variable          Matched | Treated Control    %bias  |bias| |    t    p>|t| |  V(C)

--------------------------+----------------------------------+---------------+----------

age_1                   U | 3.2447   3.3311    -13.9         |  -6.76  0.000 |  1.34*

                        M | 3.2447   3.2219      3.7    73.6 |   1.22  0.221 |  1.15*

                          |                                  |               |

gender_1                U | 1.4053   1.3369     14.2         |   6.59  0.000 |  1.08

                        M | 1.4053   1.3821      4.8    66.0 |   1.64  0.102 |  1.02

                          |                                  |               |

activity_1              U | 1.6319   1.4914     28.0         |  12.46  0.000 |  0.86*

                        M | 1.6319   1.6238      1.6    94.3 |   0.56  0.574 |  0.93

                          |                                  |               |

employ_1                U | 1.2308   1.3954    -17.5         |  -8.77  0.000 |  1.56*

                        M | 1.2308   1.2591     -3.0    82.8 |  -1.00  0.316 |  1.34*

                          |                                  |               |

edu_1                   U | 3.1839   3.4718    -26.2         | -12.40  0.000 |  1.20*

                        M | 3.1839   3.1877     -0.3    98.7 |  -0.12  0.905 |  1.21*

                          |                                  |               |

staciv_1                U | 2.9797   2.7423     13.5         |   6.35  0.000 |  1.16*

                        M | 2.9797   2.9527      1.5    88.6 |   0.52  0.606 |  1.04

                          |                                  |               |

hsize                   U | 2.6538   2.3872     21.4         |   9.93  0.000 |  1.07

                        M | 2.6538   2.6517      0.2    99.2 |   0.06  0.955 |  0.96

                          |                                  |               |

regione                 U | 11.594   9.2646     41.0         |  19.28  0.000 |  1.15*
```

```
                  M  | 11.594    11.57      0.4    99.0 |   0.14  0.888 |  1.05
                     |                                  |               |
----------------------------------------------------------------------------------------
* if variance ratio outside [0.92; 1.08] for U and [0.92; 1.08] for M
```

```
-----------------------------------------------------------------------------------
 Sample     | Ps R2   LR chi2   p>chi2   MeanBias   MedBias      B      R     %Var
-----------+-----------------------------------------------------------------------
 Unmatched | 0.074   1076.09    0.000      22.0      19.5      73.4*   1.06     75
 Matched   | 0.001      5.77    0.673       1.9       1.6       7.0    1.10     38
-----------------------------------------------------------------------------------
* if B>25%, R outside [0.5; 2]
```

References

1. Istat—Istituto Italiano di Statistica. *Indicatori Demografici Stime per l'anno 2015*; Istituto Nazionale di Statistica: Rome, Italy, **2016**.
2. Istat—Istituto Italiano di Statistica. *Conti Economici Territoriali Anno 2015*; Istituto Nazionale di Statistica: Rome, Italy, 2016. https://www.istat.it/it/files//2016/12/Conti-regionali_2015.pdf [access Feb. 25 2022].
3. Censis. *50° Rapporto Sulla Situazione Sociale del Paese*; Censis: Rome, Italy, **2016.**
4. Presidenza del Consiglio dei Ministri. *Segreteria della Conferenza Permanente per i Rapporti tra lo Stato, le Regioni e le Province Autonome*; Presidenza del Consiglio dei Ministri: Rome, Italy, **2015**.
5. Corte dei Conti (Italian Court of Accounting). *Rapporto 2016 sul Coordinamento della Finanza Pubblica (2016 Report on Public Finance Coordination in Italy)*; Corte dei Conti (Italian Court of Accounting): Rome, Italy, **2016.**
6. Ministero dell'Economia e delle Finanze (Italian Ministry of Finance). *Analisi Statistiche—Dichiarazioni 2015*; Ministero dell'Economia e delle Finanze: Rome, Italy, **2015.**
7. Budd, E. The creation of a microdata file for estimating the size distribution of income. *Rev. Income Wealth* **1971**, 17, 317–333.
8. Serafino, P.; Tonkin, A. *Statistical Matching of European Union Statistics on Income and Living Conditions (EU-SILC) and the Household Budget Survey*; Statistical Working Papers; Publications Office of the European Union: Luxembourg, **2017**.
9. Sisto, A. *Propensity Score Matching: Un'applicazione per la Creazione di un Database Integrato ISTAT-Banca d'Italia*; Working Paper n°63; Università del Piemonte Orientale: Vercelli, Italy, **2006**.
10. ISTAT. *Indagine Sulle Spese Delle Famiglie (Italian Households Budget Survey)*; Istituto Nazionale di Statistica: Rome, Italy, 2015.
11. ISTAT. *IT-SILC (Statistics on Income and Living Conditions)*; Istituto Nazionale di Statistica: Rome, Italy, 2015.
